# Evaluation of telomere length and telomerase activity on predicting in vitro fertilization treatment outcomes

**DOI:** 10.1007/s10815-024-03117-6

**Published:** 2024-05-02

**Authors:** Persefoni Fragkiadaki, Elisavet Kouvidi, Anna Angelaki, Dimitra Nikolopoulou, Elena Vakonaki, Aristidis Tsatsakis

**Affiliations:** 1 Laboratory of Toxicology and Forensic Sciences, Medical School, University of Crete, Voutes, 71003 Heraklion, Greece; 2 Lifeplus S.A., Science &Technological Park of Crete, C Building, Vassilika Vouton, 70013 Heraklion, Crete Greece; 3 Phenotypos Lab, Katehaki 40A, 115 25, Athens, Greece

**Keywords:** In vitro fertilization, Telomere length, Telomerase activity, Assisted reproductive technology, Infertility

## Abstract

The current article is a literature review aiming to provide an overview of the existing knowledge on the association between telomere length and telomerase activity and in vitro fertilization. Recently, telomeres have been used as an effective biomarker to determine biological age, which may differ from chronological age due to genetic, lifestyle, and environmental factors. Cellular senescence, along with other exogenous and mainly environmental factors, can enhance telomere wear, further shortening their ends and may also affect reproductive aging. IVF is a common fertility treatment caused by female reasons (age, ovulation disorders, damaged or blocked fallopian tubes, endometriosis), male reasons (low sperm quantity or quality), or unexplained infertility. A growing number of studies have proposed a relationship between telomere length and telomerase activity and IVF success and have suggested their use as candidate biomarkers for IVF outcome. Nevertheless, additional studies are necessary to be conducted, in order to clarify the possible implication of telomeres in IVF and to evaluate their possible role as valuable predictors of IVF result.

## Introduction

Telomeres are specialized nucleoprotein structures found at the end of linear chromosomes. In humans, telomeres are composed of tandem TTAGGG repeats bound by a specialized protein complex known as shelterin. They protect the chromosomes from damage and play a fundamental role in maintaining genomic stability and integrity. During cell division DNA is replicated to generate new cells. In somatic cells, due to incomplete DNA replication mechanism, telomeres gradually shorten with age until they reach a critical length, leading to cell cycle arrest, cellular senescence, and progressively cell death [1]. However, in germ cells, granulosa cells, early embryos, stem cells, and various types of cancerous cells, the enzyme telomerase counteracts telomere shortening, helping in the maintenance of genetic information [2]. Telomere length (TL) has been recognized as a powerful biomarker of biological aging and age-related diseases [3]. Since biological aging is strongly associated with reproductive aging, extensive research has focused on the possible implication of telomeres in human reproduction [4, 5].

In the last decades, people decide to have offspring at advanced age, mostly due to increased life expectancy, late marriages, shifting of personal priorities towards building a career rather than a family, widespread use of contraception, and other factors [6]. The combined increased average maternal and paternal age has a negative impact on fertility and reproductive capacity. Thus, many couples who face difficulties in conception turn to assisted reproductive technology (ART) treatment in order to have a baby, *with *in vitro* fertilizati*on (IVF) being the most common form [6]. IVF is a technique in which oocytes retrieved after ovarian stimulation are fertilized by sperm in a petri dish, outside the human body, and then fertilized embryos are transferred into the uterus at the cleavage or the blastocyst stage. The number of fertilized embryos transferred depend on the embryo stage and quality, maternal age, and patient preference [7]. Several factors have been associated with IVF outcome, including parental characteristics (age, gamete quality, infertility, pathologies), ovarian sensitivity, fertilization rate, embryo quality, number of transferred embryos, endometrial thickness, infertility, lifestyle factors, and culture conditions [8, 9]. One of the important stages in the IVF is the evaluation, identification, and transfer of high-quality embryos, in order to increase the probability of implantation and pregnancy outcome [8, 9]. Growing evidence links altered telomere biology with female and male infertility and pathologies of the female reproductive system [4, 5]. Recently, it has been proposed an emerging role of telomeres on embryological parameters and on IVF [4, 5]. The purpose of the present article is to further elaborate on the relationship between telomeres and IVF and to evaluate whether TL could serve as a candidate prognostic biomarker of IVF success.

## Telomeres and telomerase in female cells and their role in IVF

Oocyte is the gamete involved in critical and complex biological processes, including remodeling, in order to accept and integrate the male genome, nuclear reprogramming to totipotency in the zygote, and generalized embryonic genome activation (EGA), essential for the acquisition of its developmental competence [10]. Oocyte developmental competence is the ability of a mature female gamete to be fertilized and sustain embryo development until the blastocyst stage and ultimately pregnancy [10]. Developmental competence of this unique, highly specialized cell is promoted by the continuous interactions between oocyte germ cells and somatic surrounding granulosa or cumulus cells, in the ovarian follicle [10]. Thus, the selection of the oocyte with the best quality is of great importance for IVF result. Studies focused on the relation between telomere length and telomerase activity and oocytes are presented in Table 1.

**Table 1:** Studies focused on the relation between telomere length/telomerase activity and oocytes

Studies	Samples	Methodology used for TL/TA measurement	Main findings
Liu (2007) [14]	Fertilized eggs, embryo culture of mice	Q-FISH, q-PCR for TL/TRAP for TA	- Oocyte telomeres lengthen remarkably during early cleavage development following fertilization through a recombination-based mechanism - From the blastocyst stage onward, telomerase only maintains the TL established by this alternative mechanism
Turner (2010) [15]	4 GV oocytes and 23 preimplantation embryos	Q-FISH for TL	TL demonstrated stage-specific variations and significant differences between oocytes, cleavage stage embryos, and blastocysts
Turner (2013) [16]	Semen from 50 men and oocytes from 32 women undergoing IVF	Q-FISH for TL	- Male-derived telomeres are shorter on average than female-derived telomeres at fertilization - Human oocyte telomeres shorten during maturation
Keefe (2003) [17]	43 unfertilized human eggs after IVF	Q-FISH for TL	TL provided a better predictor of outcome following IVF than other parameters
Keefe (2005) [18]	43 GV oocytes from 21 patients undergoing IVF	Q-FISH for TL	Oocyte TL negatively predicted cytoplasmic fragmentation in D3 preimplantation embryos
Treff (2011) [19]	9 euploid/9 aneuploid polar bodies and 14 euploid/10 aneuploid blastomeres from 9 IVF patients, 10 euploid/10 aneuploid trophectoderm biopsies from sibling blastocyst stage embryos from 10 IVF patients	q-PCR for TL	Telomere DNA length was associated with human aneuploidy development
Wang (2023) [20]	120 cryopreserved human blastocysts	q-PCR for TL/TRAP for TA	Aneuploid blastocysts have longer telomeres but lower TA when compared to either euploid or mosaic/segmental human blastocysts
Turner (2019) [21]	82 first polar bodies from 25 IVF women and 86 cleavage stage (D3) embryo biopsies from 22 IVF women	q-PCR for TL	Telomere shortening was not related to chromosome segregation errors in the cleavage stage embryo
Wang (2014) [23]	Luteinized GCs from 76 women undergoing their first IVF cycle (29 pregnant and 47 non-pregnant women)	q-PCR for TL/TRAP for TA	TA is a better predictor of pregnancy outcomes following IVF treatment than T
Chen (2011) [24]	Luteinized GCs from 56 women who underwent IVF	TRAP for TA	- TA levels in luteinized GCs may predict the clinical outcomes of IVF treatment - Higher TA levels were positively correlated with clinical IVF outcomes
Cheng (2013) [25]	CCs from 45 young patients (< 38 years old) and 35 older patients (≥ 38 years old) from 80 IVF cycles	q-PCR for TL	TL in CCs is related to oocyte quality and embryo development
Yu (2022) [26]	Leukocytes and GCs from 110 women undergoing IVF divided into two age groups (< 38 and ≥ 38 years old)	q-PCR for TL	No correlation of LTL with GCTL and no predictive value of LTL for aneuploidy rate in IVF cycles
Hanson (2021) [27]	Leukocytes and CCs from 175 infertile women undergoing IVF	q-PCR for TL	Shorter LTL was associated with increasing patient age and higher rates of embryonic aneuploidy
Pentek (2023) [28]	GCs and follicular fluid from 102 patients undergoing IVF	Absolute Human TL q-PCR Assay kit for TL/TA q-PCR Assay kit for TA	No direct association of telomere function and reproductive potential, while oxidative DNA damage adversely affected early markers of IVF outcome and clinical pregnancies
Butts (2009) [29]	GCs from 54 young women (≤ 37 years old) receiving IVF (12 with occult ovarian insufficiency and 42 controls)	q-PCR for TL/TRAP for TA	In young women, abnormal telomere homeostasis is linked to concealed ovarian insufficiency
Li (2017) [30]	GCs from 163 women (65 with POCS) and 98 controls) having IVF	Fluorescence q-PCR for TL/TRAP for TA	- Lower TA and shorter TL in the GCs were linked with PCOS and a longer period of infertility - No prognostic value for the TA and TL in terms of embryo quality and IVF outcomes in PCOS patients
Xu (2017) [31]	Leukocytes and GCs from 120 individuals with biochemical POI and 279 healthy women	q-PCR for TL/TRAP for TA	- Shorter LTL and GCTL in women with biochemical POI compared with healthy controls - Diminished GCTA in patients with POI compared with healthy controls
Czamanski-Cohen (2015) [33]	Leukocytes from 20 women undergoing IVF and 10 healthy controls	q-PCR for TL	Women undergoing IVF had statistically significant higher CFD levels and shorter telomeres compared to healthy controls
Li Piani (2021) [34]	Leukocytes from 181 women undergoing IVF	q-PCR for TL	No clear link between the parameters studied and the likelihood of IVF birth

*TL*: telomere length, *TA*: telomerase activity, *CCs*: cumulus cells, *Q-FISH*: q*uantitative fluorescence* in situ hybridization, *qPCR*: quantitative polymerase chain reaction, *TRAP*: telomeric repeat amplification protocol, *IVF*: in vitro fertilization, *GV*: germinal vesicle, *D3*: day 3, *GCs*: granulosa cells, *CCs*: cumulus cells, *LTL*: leukocyte telomere length, *GCTL*: granulosa cell telomere length, *GCTA*: granulosa cell telomerase activity, *POCS*: Polycystic Ovary Syndrome, *POI*: primary ovarian insufficiency, *CFD*: cell-free DNA

Advanced maternal age (AMA > 35 years old) has a negative impact on both oocyte competence and IVF success [11]. It is widely accepted that women in their mid-30 s have all their organs and tissues work at near peak capacity, but around 40 s, there is a gradual decrease of ovarian reserve and oocyte quality and many experience infertility [11]. Within the oocyte the nucleus is more sensitive to aging than the cytoplasm and this age-related infertility has been associated with enhanced aneuploidy rates, impaired meiotic spindles, abnormal cohesin formation, and missegregation of chromosomes in the oocytes and early embryos [11, 12]. However, the exact mechanisms involved and their impact on oocyte and embryo quality are not clearly elucidated [11].

**Fig. 1:**
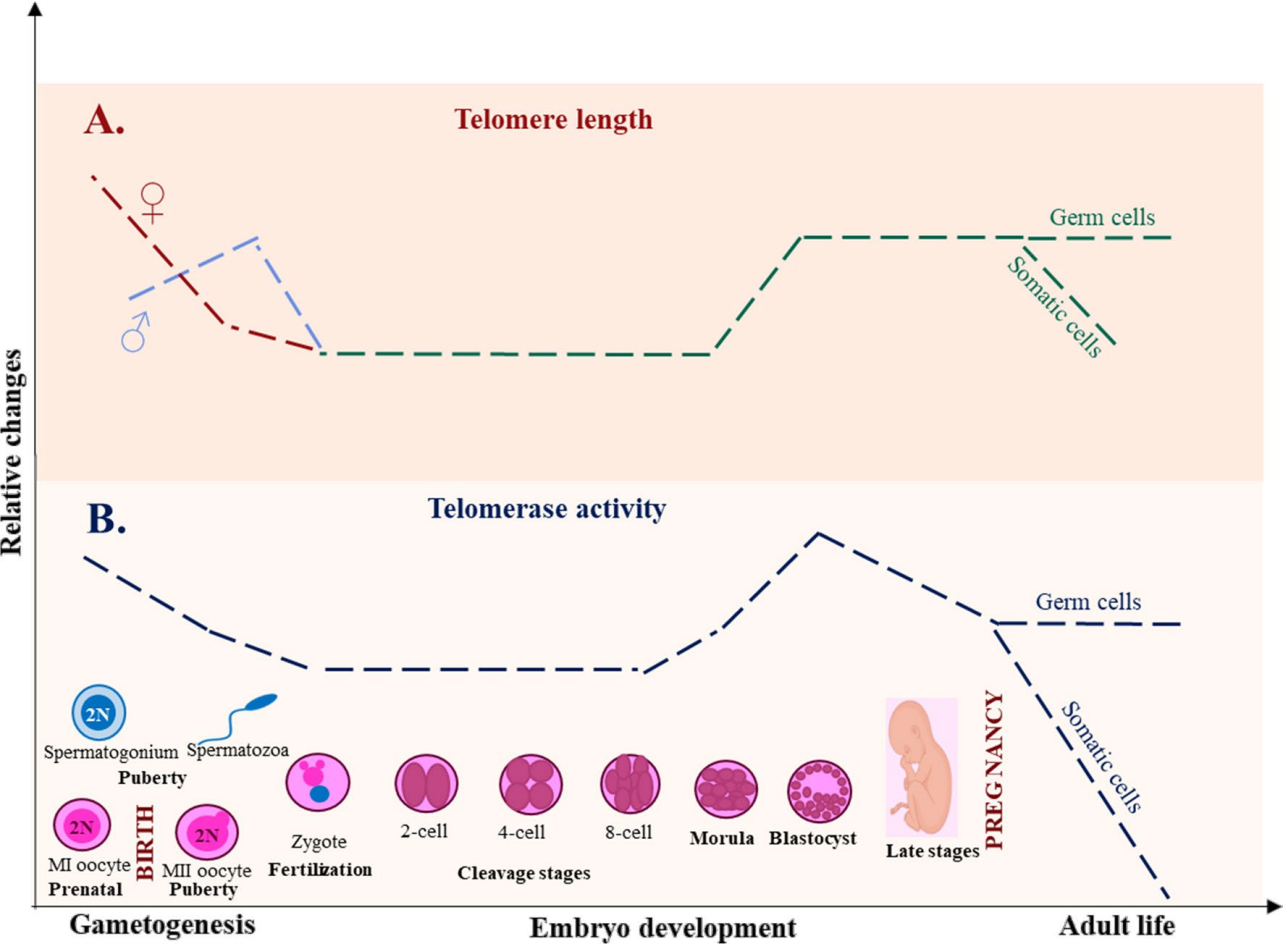
**A** Telomere length relative changes during gametogenesis, embryo development, and adult life show differences between female and male germ cells. The blue line corresponds to male germ cells (♂), the red line corresponds to female germ cells (♀), and the green line corresponds to both female and male germ cells (♀/♂). **B** Telomerase activity relative changes during gametogenesis, embryo development, and adult life show the same trend between female and male cells

It has been shown that AMA is a major contributor of dynamic shortening and dysfunction of ovarian telomeres during oogenesis and after fertilization [5, 13–16]. In oogenesis, telomerase activity (TA) is known to be relatively high in the early fetal stages and reduces during the late stages in adult ovary, indicating that telomere length is longer in the immature oocytes (11.41 ± 0.81 kb) and progressively declines through maturation, establishing the final shorter TL in the mature oocytes (8.79 ± 0.86 kb) [5, 13]. At fertilization, the female micro-environment and the interaction between oocyte and sperm telomere material determine embryonic telomere length [13, 15, 16]. During different stages of preimplantation embryonic development, telomeres are shorter in the early cleavage stages (including 2–4-cell and 5–8-cell embryos) and begin to elongate by the telomerase independent Alternative Lengthening of Telomeres (ALT) mechanism until the blastocyst stage, where they become significantly longer and it is believed to be the main point at which telomere length becomes established [14, 15] (Fig. 1).

The first observation that telomeres are implicated in IVF came by Keefe et al. who measured TL from spare eggs and their associated first polar bodies donated from women undergoing IVF and found that women who became pregnant after IVF had longer oocyte telomere lengths compared with those who failed to become pregnant [17]. Moreover, they noticed that oocyte TL less than 6.32 could be a poor predictor of a pregnancy [17]. A few years later, Keefe et al. reported that oocyte TL from 21 women who underwent IVF negatively predicted cytoplasmic fragmentation in day 3 preimplantation embryos [18]. These two studies led them propose the ‘‘Telomere theory of reproductive senescence in women,” according to which oocytes from older women have shorter telomeres because of late exit from the oogonial ‘‘production line,’’ during fetal life followed by prolonged exposure to reactive oxygen in the adult ovary [18]. Consequently, telomere shortening leads to reduced numbers of chiasmata, abnormal meiotic spindles in oocytes, aneuploidy predisposition, genomic instability, cell cycle arrest, apoptosis, and reproductive senescence in women [18].

Other studies have also tried to assess whether there is a correlation between TL and the quality of embryos from women undergoing IVF treatment. Treff et al. found that aneuploid polar bodies and cleavage blastomeres displayed significantly lower quantities of telomere DNA than paired sibling euploid polar bodies and blastomeres while Wang et al. demonstrated that aneuploid blastocysts had longer telomeres but decreased telomerase activity compared to euploid or mosaic/segmental human blastocysts [19, 20]. However, Turner et al. showed that AMA did not affect TL in cleavage stage embryos and that relative telomere length was not altered between aneuploid and euploid first polar bodies [21]. In addition, they revealed that telomere shortening was not associated with chromosome instability in the cleavage stage embryo and they suggested no evidence of the telomere theory of reproductive ageing in relation to aneuploidy generation [21].

Some studies have investigated TL and telomerase activity in oocyte’s surrounding somatic cells, known as granulosa (GCs) or cumulus (CCs) cells. These cells are crucial for oocyte quality, since they are in close communication with them and play a critical role in regulating their growth, maturation, ovulation, and fertilization [22]. Moreover, the development of GCs is synchronized with that of oocytes, and it can indirectly reflect the development of oocytes [23]. Chen et al. found that TA levels in luteinized GCs may participate in the regulation of their function, and thus may indirectly influence the activation of oocytes [24]. Additionally, they suggested that higher levels of TA in GCs were positively correlated with clinical outcomes of IVF treatment [24]. Wang et al. demonstrated that in GCs, TA, rather than TL, is a better predictor of pregnancy outcomes [23]. Cheng et al. reported that relative TL in CCs negatively correlated with subjects’ ages [25]. Additionally, they observed that shorter telomeres in CCs derived from immature oocyte, unfertilized eggs, and poor-quality embryos and they suggested that the relative TL in human CCs is important for oocyte maturation and embryo development [25]. Yu et al. demonstrated that TL in the granulosa cells was negatively correlated with the aneuploidy rate in the young-age group, supporting the application of Preimplantation Genetic Testing for Aneuploidy (PGT-A) in younger women [26].

However, data from a study analyzing TL of CC samples noted no significant associations between CC relative telomere length and any outcomes assessed [27]. Recently, Pentek et al. could not confirm a direct association between telomere function of GCs and follicular fluid (FF) and reproductive potential, while oxidative DNA damage, particularly in FF, adversely affected markers of IVF outcome and clinical pregnancies [28]. Consequently, the use of both GCs and CCs as potential biomarkers of oocyte and embryo quality warrants further clarification. Experiments on the association between telomeres and IVF outcomes have also been performed in women with fertility pathologies. Butts et al. reported that GC telomeric shortening and diminished telomerase activity were associated with occult ovarian insufficiency and lower clinical pregnancy rates, higher miscarriage rates, and lower live birth rates [29]. Li et al. indicated that lower TA and shorter TL in the GCs were associated with Polycystic Ovary Syndrome (PCOS) and a longer duration of infertility, while no predictive value was found for the TA and TL in terms of embryo quality and IVF outcomes in PCOS patients [30]. Xu et al. showed that TL was shortened with advancing age and that women with biochemical primary ovarian insufficiency (POI) had shorter GCTL and diminished telomerase activity compared with healthy controls [31]. However, no differences in rates of cleavage and high-quality embryo on day 3 (D3) were observed between shorter and longer GCTLs either in patients or in controls [31].

Because of the difficulties to obtain oocytes and embryos and the observation that age-related TL shortening occurs in most tissue types of an individual, measurements of TL from peripheral blood leukocytes (LTL) have been used as an alternative for predicting IVF outcome [32]. Czamanski-Cohen et al. revealed that women undergoing IVF had shorter LTL compared to healthy controls, but they did not examine IVF outcome [33]. Xu et al. in their previous study also measured LTL and found shorter LTL in women with POI associated with advancing age, but no difference in IVF parameters (rates of cleavage and high-quality embryo on D3 in TL between patients and controls) [31]. Hanson et al. reported that shorter LTL of women undergoing IVF was associated with increasing patient age and higher rates of embryonic aneuploidy [27], while Li Piani et al. failed to show a marked association between LTL and the chances of live birth in IVF [34]. These contradictory results are due to the selection of samples and the methodologies used for the measurement of TL and TA.

## Telomeres and telomerase in male cells and their role in IVF

It is now well established that the fertilizing spermatozoon contributes numerous factors which interact with their female counterparts and has a dynamic and critical participation in normal fertilization and postfertilization developmental steps [35]. A deeper comprehension of the contributions of paternal components and the assurance of sperm quality are crucial for ART success [35]. Thus, it is of great importance the identification of a novel diagnostic and prognostic biomarker for the evaluation of sperm quality and function [36]. Accumulating data have demonstrated that sperm TL (STL) is a new molecular marker of sperm quality [36]. Studies focused on the relation between telomere length and telomerase activity and spermatogenic cells are presented in Table 2.

**Table 2:** Studies focused on the relation between telomere length/telomerase activity and spermatogenic cells

Studies	Samples	Methodology used for TL/TA measurement	Main findings
Kimura (2008) [50]	Leukocytes from 634 men (18–94 years old) and sperm from 46 young (< 30 years) and older (50 years) donors	Q-FISH	- LTL in offspring was positively correlated with paternal age at the time of birth - A subset of sperm in older men had with elongated telomeres
Antunes (2015) [49]	20 individual spermatozoa from 10 men undergoing IVF	q-PCR	TL among individual spermatozoa within an ejaculate varies markedly and increases with age
Mishra (2016) [37]	112 infertile men and 102 age-matched fertile controls	q-PCR	Mild oxidative stress results in longer telomeres, while severe oxidative stress results in shorter telomeres
Gentiluomo (2021) [43]	599 men undergoing semen evaluation	q-PCR	- No associations between sperm parameters and STL nor LTL were observed - Four SNPs were weakly associated with sperm variables
Cariati (2016) [44]	73 samples from men (31–52 years old)	q-PCR	STL was found to be strongly related to sperm count, implying the theory that aneuploidy and sperm DNA fragmentation are linked
Biron-Shental (2018) [45]	100 sperm cells from 16 sub-fertile and 10 fertile men	Q-FISH	Sub-fertile sperm cells have short telomeres that are elongated by the alternative pathway of telomere capture
Berneau (2020) [51]	66 normozoospermic male partners of couples undergoing ART	q-PCR	STL is related to IVF rates but not to sperm characteristics or lifestyle factors
Sharqawi (2022) [52]	34 men (18–60 years old) from couples undergoing IVF	q-PCR	A healthy lifestyle is connected with extended STL and good sperm quality in men undergoing IVF
Yang (2016) [56]	306 overweight males (> 28 kg/m^2^) and 345 age-matched normal weight individuals (20–25 kg/m^2^)	q-PCR	- Couples with male BMIs > 28 kg/m^2^ had poorer rates of fertilization, superior embryo development, and clinical pregnancy rate than couples with normal BMI males - Mean STL value for the overweight BMI group was significantly shorter than that of the normal BMI group
Yang (2015) [57]	418 men from couples planning IVF	qPCR	STL was positively correlated with embryo quality and transplantable embryo rates, but not with clinical pregnancy rates

*TL*: telomere length, *TA*: telomerase activity, *Q-FISH*: quantitative fluorescence in situ hybridization, *LTL*: leukocyte telomere length, *IVF*: in vitro fertilization, *STL*: sperm telomere length, *q-PCR*: quantitative polymerase chain reaction, *SNP*: single-nucleotide polymorphism, *ART*: assisted reproductive technology, *BMI*: Body Mass Index

Numerous studies have discovered a favorable relationship between STL and traditional indicators of high sperm quality, such as motility, vitality, and sperm count. This has also been linked to other DNA integrity-related traits, with research showing a correlation between STL and DNA fragmentation and protamination levels. Additionally, it is important to mention that telomere attrition, which is mostly linked to an oxidative stress situation, influences sperm quality and has a negative impact on reproductive potential and increased DNA instability [37].

Experiments have demonstrated that there is a link between sperm DNA damage and semen characteristics, male infertility, conception, and treatment response [38, 39]. Telomere length also seems to be a reliable biomarker for female and male infertility since it has a significant role in fertilization outcomes at in vitro setting [40]. Vasilopoulos et al. provided an outline of general trends regarding the association of TL with infertility factors, by proving epidemiological and original research studies [41]. A favorable association between male infertility factors and shorter STL was found in the majority of the studies [41]. Amir et al. revealed that the death of spermatozoa, decreased motility, low sperm count, incorrect chromosomal pairing and movement during meiosis, and unsuccessful fertilization are some of the mechanisms through which sperm telomere shortening is linked to male infertility [42]. However, Gentiluomo et al. did not find a direct correlation between telomere and male infertility in their analyses on male spermatogenesis and infertility [43]. They found four single-nucleotide polymorphisms (SNPs) to be weakly correlated to sperm variables, suggesting that these SNPs are pleiotropic and may be involved in other regulatory mechanisms unrelated to telomere homeostasis but nonetheless involved in the spermatogenic process [43]. The findings of their research indicate that while TL is not directly associated with male infertility, the selected SNPs may still be involved indirectly [43]. In order to determine if STL variation is related to chromosomal abnormalities, DNA fragmentation, conventional semen characteristics, IVF success, or all four factors, Cariati et al. analyzed STL in semen samples from men [44]. They showed that shorter telomeres are correlated with an elevated percentage of diploidy [44]. Oligospermic samples exhibited particularly short telomeres, and STL was also discovered to be positively linked with sperm count [44]. Atypical STLs were present in 17.6% of the samples in total [44]. All these samples failed to result in a continuing pregnancy [44]. They concluded that STL has the potential to be a quick and affordable method of evaluating sperm quality [44]. Moreover, according to additional studies, males who have idiopathic infertility have significantly shorter sperm telomeres and a lack of telomere homeostasis than healthy controls, which is demonstrated by lower amounts of telomerase [44]. Similar findings were made by Biron-Shental et al. who discovered that subfertile men had less sperm cells with telomerase reverse transcriptase positivity and more sperm cells with shorter TL than controls [45]. Early spermatogenesis has been associated with elevated telomerase expression, whereas spermiogenesis, the late phase of spermatogenesis, is associated with falling telomerase expression (Fig. 1). This suggests that whereas spermatids and mature spermatozoa exhibit decreased telomere lengths, spermatocytes preserve their telomere length [45]. Another possibility is that numerous complex chromosome reorganization processes take place during the late stages of spermatogenesis [5]. Thus, an altered telomere dynamic in sperm may be a possible cause for a decline in male reproductive potential, especially in idiopathic instances, and points to the detrimental effects of telomere shortening on sperm function [46].

Spermatogenesis is a dynamic and continuous process in men with no critical age at which sperm production cease [42]. However, advanced paternal age (APA) has been shown to negatively affect sperm quality and testicular functions [47]. In addition, APA has been associated with epigenetic changes, DNA mutations, chromosomal abnormalities, increasing rate of preterm death, and decreased IVF success rate [47]. It is well known that STL increases with advancing age maybe due to the high telomerase activity in the testes and/or the ALT pathway [48]. Male germ cells have average TL 10–20 kb, with elongation ranging from 17 to 135 bp/year depending on the methodology used [36, 42]. TL among individual sperm subpopulations within an ejaculate also show increasing heterogeneity with advancing age [49]. Moreover, APA at conception has been positively correlated with longer LTL and STL in offspring of older fathers, thus confirming the telomere length heritability [36, 48, 50].

Selection of spermatozoa with long telomeres is essential for the development of excellent quality embryos, and may even be necessary for a successful IVF outcome, according to several studies that link STL with good quality embryos [5, 51–53]. Specifically, spermatozoa with longer telomeres are more likely to result in better quality embryos and possibly to a successful IVF outcome [5]. ΙVF failure and a higher likelihood of recurrent miscarriage have been linked to embryo aneuploidy, with short telomeres thought to be the primary cause of aneuploidy and delayed embryo development [5]. These evidences are endorsed by further research that showed that in normozoospermic samples, there was a positive link between sperm telomere length and fertilization rate, as well as a clear trend towards higher sperm telomere length in successful embryo implantation rates [51]. However, no link between sperm telomere length and sperm parameters was discovered and no correlation was found between sperm telomere length and lifestyle factors [51]. Overall, the findings imply that sperm telomere length may have an essential mechanistic role in fertilization rate regardless of sperm characteristics or lifestyle factors [51]. On the contrary, Sharqawi et al. examined how lifestyle choices affected sperm’s telomere length and tracked how that relationship related to IVF success [52]. They found that a good lifestyle is associated with long STL and good sperm quality in patients undergoing IVF [52]. In addition, Yuan et al. performed a meta-analysis, including 12 prospective observational cohort studies in order to assess the accuracy and clinical value of STL as a new marker for diagnosing male infertility and predicting the quality of embryonic development [53]. They revealed that embryonic aneuploidy may be linked to an elevated risk of IVF failure and recurrent miscarriage [53]. Given the fact that short telomeres are correlated with increased aneuploidy and delayed embryo development, it was hypothesized that sperm telomere length might be a promising predictor of embryonic development, both for natural conception and IVF, because it could represent embryonic quality and predict pregnancy outcome to some extent [53].

Interestingly, Van Opstal et al. displayed that in couples undergoing IVF therapy advanced male age adversely influences the likelihood of reaching the 8-cell stage at D3 [54]. They observed a significant inverse association between APA and a key determinant for scoring of embryo quality: older men were less likely to produce an embryo of eight blastomeres at D3, compared to younger fathers [54]. On the other hand, Lu et al. did not discover a relationship between male age and high-quality or transferrable embryos, instead they revealed that blastocyst formation rates were unaffected by male paternal age and normal semen characteristics [55]. In the same research, it was indicated that there were no differences in the birth weights of newborns across the various paternal age groups, and that the miscarriage rate for fathers between the ages of 35 and 39 was substantially higher than that for fathers under 35 [55]. The live birth rate was on the decline, although there was no appreciable variation between groups [55]. Yang et al. studied the effect of paternal overweight or obesity on IVF treatment outcomes and the possible mechanisms that are involved. They supported that couples with males who had Body Mass Index (BMI) exceeding 28 kg/m^2^ had lower rates of fertilization, high-quality embryo development, and clinical pregnancy than couples with men who had BMIs between 20 and 25 kg/m^2^ [56]. Additionally, the mean STL for each patient was calculated, and the findings revealed that the overweight BMI group’s mean value was much shorter than that of the normal BMI group [56]. Additionally, it was discovered that whereas sperm mitochondrial activity was lower in the overweight BMI group compared to the normal BMI group, sperm DNA fragmentation rate and reactive oxygen species (ROS) content in semen were higher [56]. Finally, Yang et al. found a correlation between sperm TL and the viability of IVF-created embryos, indicating that the telomeric state of male gametes may have an impact on later embryonic development [57]. To ascertain the relationships between STL, fertilization laboratory parameters, and clinical pregnancy in IVF, researchers evaluated 418 couples [57]. The mean STL for each patient was found using the quantitative PCR method after semen samples were collected [57]. They discovered that STL is positively correlated with embryo quality during IVF and suggested that STL may be utilized as a marker for the prediction of embryonic quality. However, more research is required to support these findings [57].

To conclude, even though the literature shows conflicting evidence on the effect of STL on semen quality, the majority of studies imply that STL can be an effective biomarker for male infertility and possibly help in the improvement of the success rate of fertility treatments [58]. The conflicting findings on STL in semen as a fertility marker could be due to limitations of the study, such as small size, different populations studied, and different techniques used. Thus, it is of great value for further studies to take place and define whether STL is an appropriate biomarker for male infertility and whether it could be a valuable tool for assisted reproduction [58].

## Other factors affecting IVF outcome

IVF includes 4 crucial steps: ovarian stimulation, oocyte retrieval, embryo fertilization, and embryo transfer [7]. During these procedures, gametes and embryos are exposed to numerous factors and chemicals, which may induce mitochondrial, genetic, and epigenetic alterations on TL and TA in IVF-derived gametes and embryos, with potential impact on their quality [59]. Regarding ovarian stimulation, several studies have reported that superovulation can cause alterations in the epigenome of oocyte and embryos; however, studies are needed to evaluate how ovarian stimulation protocols influence TL and TA of oocytes and embryos [59]. Oocytes retrieved are then fertilized with the collected sperm. A growing evidence demonstrates that manipulations of sperm samples during IVF (handling, separation method, washing techniques, cryopreservation) could alter paternal epigenome or sperm trancriptome and could impair fertilization, embryo quality, and IVF outcome [60, 61]. Several reports have suggested that in vitro culture period and conditions, such as culture media, oxygen levels, temperature, humidity, osmolality, and pH, which mimic human organism during IVF process, can influence implantation, as well as pregnancy rates due to their effect on embryo quality [5, 59–62]. Recently, a limited number of studies have shown that the exposure of oocytes and embryos to culture conditions may increase ROS amounts and influence intracellular events (e.g., epigenetic modifications), which may trigger changes of telomere length in the early embryos [5, 59–62]. Another interesting factor is the increased BMI in females and in combined females and males, which is associated with lower numbers of available embryos, high-quality embryos, and decreased fertilization rates in the female group with increased BMI [63]. However, only one study has shown that high maternal BMI is associated with shorter LTL and activation of telomere shortening and this was linked with a poor IVF outcome [5, 64]. Moreover, exogenous and mainly environmental factors (pollution, exposure to toxic agents, pesticides, or herbicides) may indirectly affect embryo TL and IVF outcome [5]. Additional studies are required to elucidate potential negative impact of these factors on telomeres.

## Conclusions

Mounting evidence suggests a close relationship between telomeres and telomerase and reproductive aging. Although a growing number of studies have proposed a positive association between telomeres and IVF, the data is limited and further research is needed to shed more light into their role in IVF. Given that telomere length and telomerase analysis is a reasonably simple and inexpensive approach, it may meet the criteria for its inclusion in the IVF procedure as a prognostic biomarker for determining embryo quality, as well as pregnancy success.

## Abbreviations

TL: Telomere length
ART: Assisted reproductive technology
IVF: In vitro fertilization
EGA: Embryonic genome activation
AMA: Advanced maternal age
TA: Telomerase activity
ALT mechanism: Alternative Lengthening Telomere mechanism
GCs: Granulosa cells
CCs: Cumulus cells
PGT-A: Preimplantation Genetic Testing for Aneuploidy
FF: Follicular fluid
PCOS: Polycystic Ovary Syndrome
POI: Primary ovarian insufficiency
LTL: Leukocyte telomere length
D3: Day 3
STL: Sperm telomere length
SNPs: Single-nucleotide polymorphisms
APA: Advanced paternal age
BMI: Body Mass Index
ROS: Reactive oxygen species
